# Work motivation and occupational self-efficacy belief to continue working among ageing home care nurses: a mixed methods study

**DOI:** 10.1186/s12912-021-00780-3

**Published:** 2022-01-27

**Authors:** Stina Wallin, Anncristine Fjellman-Wiklund, Lisbeth Fagerström

**Affiliations:** 1 Faculty of Education and Welfare Studies, Health Sciences, Åbo Akademi University, Strandgatan 2, 65101 Vaasa, Finland; 2 Department of Community Medicine and Rehabilitation, Physiotherapy, Umeå University, 901 87 Umeå, Sweden; 3 Faculty of Health and Social Sciences, University of South-Eastern Norway, Grønland 58, 3045 Drammen, Norway

**Keywords:** Ageing workers, Home care nurses, Occupational health, Healthy ageing, Personal resources, Occupational self-efficacy, Work motivation, Mixed methods

## Abstract

**Background:**

It is important to support ageing home care nurses (HCNs) to remain in work for longer, since the need for home care services is increasing. Personal resources such as self-efficacy belief contribute to work ability, as does work motivation. Few studies have targeted the ageing workers’ self-efficacy belief to manage their final working years. This study explores ageing HCNs’ work motivation, and occupational self-efficacy, i.e. belief in one’s capabilities, to continue working until expected retirement age.

**Methods:**

The design of the study is exploratory using a mixed method with a qualitative to quantitative approach. A total of 234 HCNs answered four open-ended questions from a cross-sectional survey, regarding their work motivation and self-efficacy beliefs. First, data was analysed using manifest qualitative content analysis. Next, a quantitative analysis was performed based on the results of the qualitative study, and the categories that emerged were quantitatively ranked.

**Results:**

The open-ended questions yielded 2339 utterances. The findings showed that several categories concurrently affected both work motivation and self-efficacy belief. When they were well-functioning, they positively affected both work motivation and self-efficacy belief, and when they were insufficient, they negatively affected either or both motivation and/or belief. Meaningfulness, job satisfaction, social support, and work environmental and organizational characteristics affected work motivation most. Perceived health highly affected the self-efficacy belief to continue working until expected retirement age, as well as meaningfulness of work, support from colleagues and home care managers, and work characteristics.

**Conclusions:**

Through highlighting the meaningfulness of work, and supporting the perceived health, the work community and leadership, both work motivation and self-efficacy belief to continue working might be facilitated among ageing HCNs. However, the still present draining workload must be handled.

**Supplementary Information:**

The online version contains supplementary material available at 10.1186/s12912-021-00780-3.

## Background

In home care, workers are the greatest resource since a lack of workers means limited service [1]. In many countries the supply of home care services has increased or aim to increase [1–3] as a consequence of that the European population is getting older, and, hence, the need for care is expected to escalate [4]. The majority of older people also prefer to remain in their homes as long as possible [1–3], where they are able to better maintain their functional ability [3]. However, an already existing challenge is the shortage of home care personnel [2, 5], and, a large percentage of whom are ageing [6, 7]. Moreover, about 40% of home care nurses (HCNs) in the Nordic countries [6, 7], and 75% in Finland [8] have considered quitting their jobs. Reasons for this are the increased physical and mental workload caused by a higher number of, more complex clients, and less personnel [3, 6, 8]. HCNs have expressed concerns about both the quality of home care and the safety of the clients [3, 6, 8], as well as their own health [6, 8]. Despite this, HCNs still experienced meaningfulness of work [6].

According to the Job Demands-Resource (JD-R) model, work characteristics can be described through the psychological processes of job demands and resources [9, 10]. Job demands describe a health-impairment process and refer to physical, psychological, social, or organizational job-related strains that require physical and/or psychological effort. Job resources describe a motivational process, and are defined as physical, psychological, social, or organizational aspects that either reduce job demands and the associated physiological and psychological costs, or are functional in achieving work goals, or stimulate personal growth, learning and development [9, 10]. In a subsequent extension of the J D-R model, personal resources have been included and described as individuals’ sense of whether they can successfully control and influence their environment [10]. Personal resources may have the same motivational potential as job resources. When personal resources are high, workers are confident about their capabilities, more resilient, and optimistic about their future [10]. Personal resources might help workers be more resistant against adverse work demands [10] and may, together with job resources, moderate the negative effects of age on work ability [11]. However, practices supporting ageing workers mostly focus on reducing job demands and increasing job resources, and seldom on enhancing personal resources [12].

The importance of HCNs’ motivation for maintaining home care services [1], and for the quality of healthcare [13] has been emphasized in previous research. Nurses’ work motivation refers to the willingness to work well and to the accomplishment of tasks and goals [13], and contributes to work ability among home care personnel [14] and nurses [13]. Nurses’ work motivation is individually created and affected by both personal internal factors and external work-related factors. Intrinsic motivation, i.e. performing an activity for its own sake in order to achieve satisfaction [15], is a key to continue working until retirement age [16]. Ageing workers seems to be more motivated by intrinsic aspects [17], such as meaning of work for example [6, 11, 16, 18, 19]. Earlier studies have stressed the importance to focus on factors that motivates ageing workers to stay in work [20], as well as on strategies enhancing intrinsic motivation [21]. Since factors influencing work motivation may differ in different cultures and contexts of nursing practice [13], we venture to say that work motivation also should be explored in the specific context of ageing HCNs.

Self-efficacy is an important personal resource, promoting work ability in home care staff [14], intrinsic motivation [22] in ageing workers [23], and to wellbeing at work [10, 24]. Occupational self-efficacy refers specifically to the occupational domain and describes belief in one’s capabilities to successfully perform the tasks involved in one’s work [25]. Previous research has stressed the importance of enhancing self-efficacy in workplace interventions, alongside job resources and job demands [14], also for older workers [23, 26]. Despite much research on self-efficacy in general, less is known about occupational self-efficacy in ageing work force [23], as well as few studies have targeted the ageing workers’ personal resources [12]. However, a recent study found that occupational self-efficacy was related to ageing HCNs’ work ability [27]. To enhance occupational self-efficacy in ageing HCNs, it is therefore important to explore what influence occupational self-efficacy belief to continue working.

In sum, this study is justified by the increasing need for home care, which includes a greater workload for the ageing HCNs, as well as the importance of work motivation and occupational self-efficacy for a long work career, which is underexplored in ageing HCNs. The generated knowledge could be valuable for retaining HCNs and attracting workers to the home care sector, and supporting good quality of care.

The aim of this mixed methods study is therefore to explore the ageing HCNs’ work motivation and occupational self-efficacy to continue working until expected retirement age.

The research questions are:

- What positively and negatively affect ageing HCNs’ work motivation and occupational self-efficacy belief to continue working until expected retirement age?

### Context of research

In many municipalities in Finland, home service and home nursing care are combined as home care [28]. Home care work is organized in accordance with the Finnish legislation, and usually entails day-to-day assistance and nursing to help older persons and individuals with diminished functional capacity, due to disabilities and illness, cope with their everyday needs and activities [28]. Home care tasks have changed in recent years because of organizational fluctuations [6]. Nowadays core services include personal care, health care, lifting and documenting, and there is less time for social support, cooking, and shopping. In many municipalities it is possible to obtain home care also in the evening, at night and on weekends [28]. Lunches and breaks are usually included in enterprise resource planning in connection with transfers between clients [8]. Home care personnel in Finland primarily consist of practical nurses and home support workers [8]. In 2015, 73% of home care personnel had more than 2 years, and 23% one to 2 years of care education (vocational level) [6]. In 2020, the expected retirement age for a 50-year-old in Finland was 63.8 years [29]. In this study, we use the term “home care” since it was commonly used in the municipalities where the study participants worked. The term “home care nurses” will be used for all personnel working in home care.

## Methods

### Study design

The design of the study is exploratory using a mixed method with a qualitative to quantitative approach [30].

### Setting, data collection and participants

The study is part of the larger research project “Personal resources supporting work ability”, aiming to provide knowledge about ageing workers’ personal resources during their final working years. In this study, we considered ageing workers 45 years and older. Forty-five years has often been used as a criterion for ageing workers since major changes in function that can affect work ability and personal resources often occur at this age; yet preventive measures are still possible [31]. The study was carried out in May to September 2018. The questionnaire included three well-reputed, valid, and reliable measuring scales and four open-ended questions, which were analysed separately in two different phases. The results from the measuring scales Work Ability Index [32], Utrecht Work Engagement Scale [33] and Occupational Self-Efficacy Scale – Short Form [25] are presented elsewhere (accepted, not yet published).

**Fig. 1:**
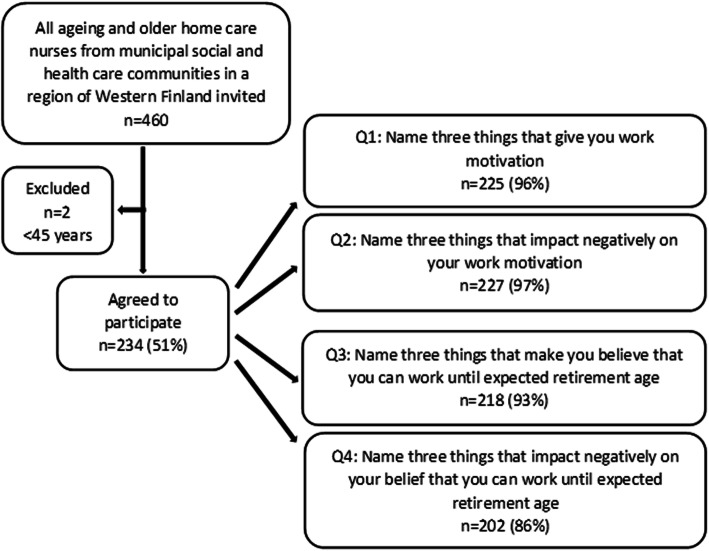
Flowchart of the study population

The HCNs’ answers from the open-ended questions composed the source of this study. An English translation of the open-ended questions is provided as Additional File 1. From a region of Western Finland with mostly rural areas, HCNs were recruited using their home care manager, that is, the manager of the home care unit. Eight municipalities and cities, conducting home care services by themselves, and two social- and health services consortiums consisting of three to four municipalities or cities each, and who all were organizing public home care in accordance with Finnish legislation, were included. All HCNs, 45 years and older, were recruited to answer an anonymous pilot tested paper-based or web-based questionnaire available in Swedish or Finnish languages. In the open-ended questions, the HCNs were asked to name three things that give work motivation and three things that impact negatively on their work motivation, and correspondingly that make them believe that they can continue working until expected retirement age and that impact negatively on their belief to continue working (i.e. occupational self-efficacy) (Fig. 1). In this study, motivation refers to willingness to work well [13], and occupational self-efficacy to belief in one’s capabilities to successfully perform work until expected retirement age [25].

Out of 460 invited participants, 234 HCNs answered the questionnaire after three reminders, yielding a response rate of 51%. Participants were 45–66 years old, and the majority were women with an average of 18 years work experience in home care (Table 1).

**Table 1:** Characteristics of the study population (*n* = 234)

Age (years)	55 (55), 5.3
45–49	19.4
50–54	26.6
55–59	31.1
60–66	23.0
Gender	
Women	99.1
Men	0.9
Marital status	
Single	8.3
Married/marriage-like relationship	80.3
Divorced	11.0
Widow	0.4
Native language	
Swedish	57.3
Finnish	42.7
Educational level	
No vocational training	2,7
Vocational degree	65,5
Higher vocational education	28,3
Master’s degree or higher education	3,5
Employment status	
Permanent employment	88.3
Temporary employment	11.7
Full-time employment	73.0
Part-time employment	27.0
Job demands	
Mentally demanding	18.5
Physically demanding	5.6
Mentally and physically demanding	75.9
Work experience (years)	18 (18), 12.1
Work ability index	38 (40), 6.8
Occupational self-efficacy	5.9 (6.0), 0.8
Work engagement	5.1 (5.3), 0.9

The numbers are either % or mean (median), standard deviation.

Work ability index: poor (7-27), moderate (28-36), good (37-43) and excellent (44-49) work ability. Occupational self-efficacy: 0–7, higher mean scores indicate better self-efficacy. Work engagement: 0–6, Higher mean scores indicate better work engagement.

### Data analysis

A mixed methods approach was used, including qualitative and quantitative research [30, 34]. Using mixed methods enables a better understanding of the complex topic, using the strength of integrating both qualitative and quantitative analyses to generate new knowledge. First, the answers from the open-ended questions were analysed by qualitative manifest content analysis, to gain a deeper knowledge and understanding [30, 35] of ageing HCNs’ work motivation and occupational self-efficacy belief. Answers from the open-ended questions were organised in Microsoft Excel, with answers from each participant in a separate row. To become familiar with the text, all responses were read through several times. From each question, responses were grouped into topics. Further, for each individual question, the topics were analysed by manifest qualitative content analysis. The condensed text was coded, and the codes were interpreted and compared repeatedly for similarities and differences. Codes with similar content were sorted into sub-categories and categories. Finally, sub-categories were sorted into categories that corresponded to the meaning of the material, the context, and the aim. To confirm conformability, investigator triangulation was used. SW made the initial analysis of the responses into similar topics and condensed the text. In addition to SW, AFW and LF read the condensed answers and the codes independently, and the codes were compared and discussed until agreement was reached [30, 35]. Next, descriptive statistics were performed using Microsoft Office 2016 Excel, based on the results of the qualitative analysis. The categories created from the qualitative analysis were quantitatively ranked, based on the frequencies of which they were mentioned in all the data for each open-ended question [30]. Reporting the frequencies and percentages enabled examination of where the main point in HCNs’ responses was located in the large amount of data (2339 utterances from the four questions taken together). An integration of both the qualitative and the quantitative approach was used, in the aim, the data analysis, results and discussion [30, 34].

## Results

**Fig. 2:**
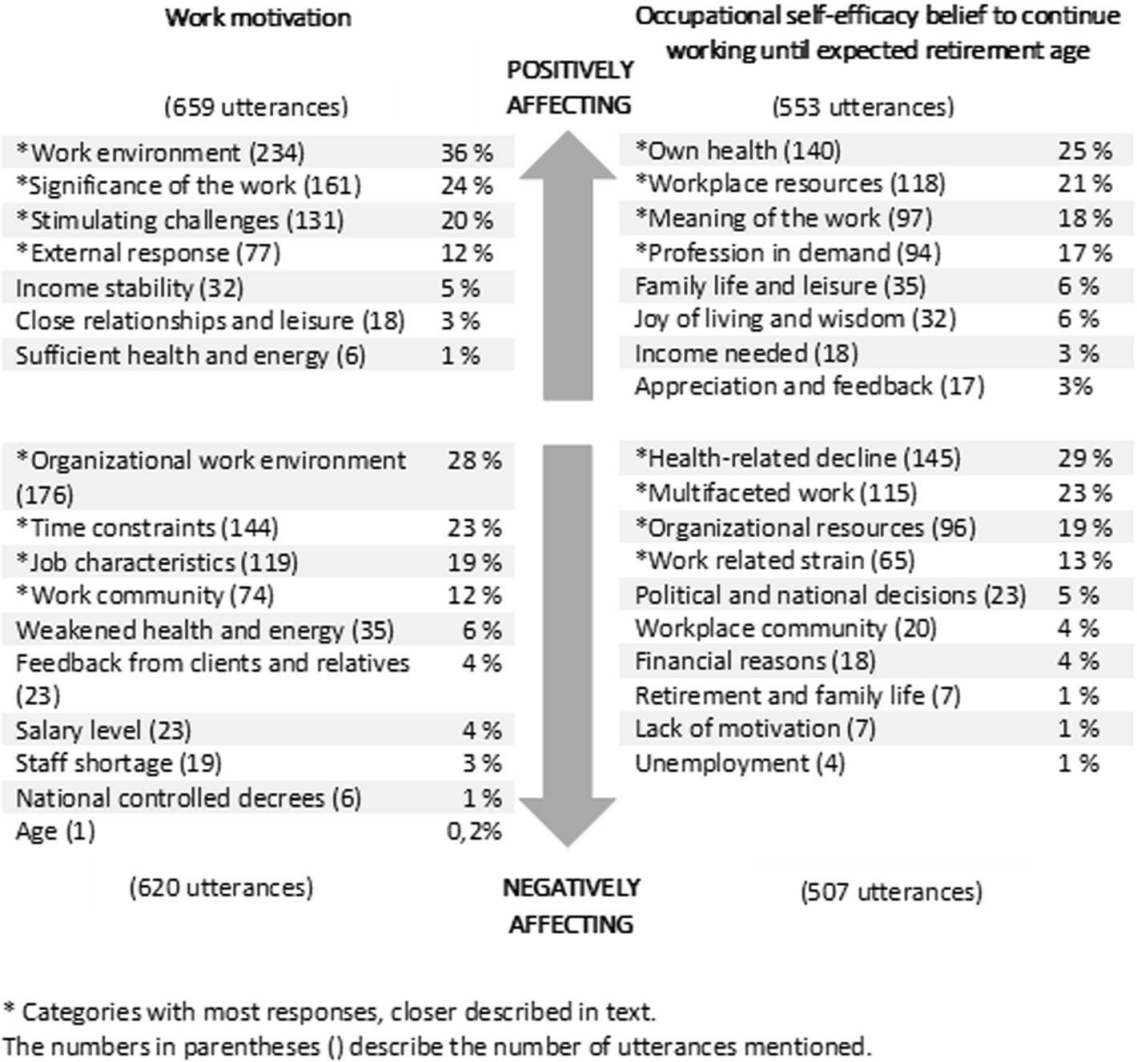
Categories positively and negatively affecting work motivation and occupational self-efficacy belief

The analysis revealed seven categories describing what gives work motivation, and 10 categories negatively affecting work motivation. Eight categories emerged describing what facilitates HCNs’ belief to continue working until expected retirement age, and 10 categories negatively affecting belief. There were 659 utterances related to categories positively affecting work motivation (*n* = 225, 96%), and 620 utterances negatively affecting work motivation (*n* = 227; 97%). Regarding what positively affect occupational self-efficacy belief to continue working until expected retirement age, 553 utterances were emphasized (*n* = 218; 93%), while 507 utterances were related to what negatively affect HCNs’ belief (*n* = 202; 86%). Typically, the participants gave between one to three responses for each question. For each individual question, the categories that gathered the most utterances, and their sub-categories, are presented more closely below. The results of the qualitative content analysis, including quotations, are presented in Table 2, and the frequencies for each question are presented in Fig. 2.

**Table 2:** Results of the qualitative content analysis

Categories	Sub-categories	Quotations
***Categories positively affecting work motivation***		
Work environment	Satisfaction with colleagues	*“being with coworkers [makes me feel good]”* *“wonderful workmates and the cooperation we have”* *“good and supportive work team”*
	Satisfaction with leadership and organizational resources	*“have influence over own work, can affect”* *“to have a good supervisor, who listens etc.”*
Significance of the work	Meaningful work	*“experience that the work is important”* *“the feeling that you make a difference in the elderly’s everyday life”*
	Enriching client meetings	*“to get to work with elderly people”* “*get so much back from the clients*”
Stimulating challenges	Multifaceted work	*“it isn’t monotonous work, something happens all the time”* *“work independently, must make own decisions”* *“challenging, requires physical and mental fitness as well as social skills”*
	Competence and desire to develop	*“I feel I can do my job”* *“opportunity to develop, learn new things”*
External response	Recognition from clients and relatives	*“gratitude from clients and relatives”* *“when a client thanks [you] for the help and is satisfied with the care”*
	Recognition from colleagues and management	*“positive feedback from coworkers”* *“appreciation from superiors”*
***Categories negatively affecting work motivation***		
Organizational work environment	Lacking resources	*“not equipment to get the job done”* *“one should constantly take in so many darn new things”* *“existing knowledge is not utilized”*
	Unsatisfactory leadership	*“if superiors don’t listen or don’t care”* *“unappreciative head nurse, only hear how bad we are”* *“poor management – don’t understand our job”*
Time constraints	Significant rush and stress	*“hurry, tight work schedules that others plan”* *“not knowing whether you have time to eat during the work day”* *“lack of time [with] the clients, just in a hurry all the time”*
	Negative consequences of time pressure	*“the lack of time - not having the time to perform one’s tasks”* *“things that are left unfinished”*
Job characteristics	A challenging work	*“change of clients, that you are not allowed to care for the same clients but are instead forced to change daily = IRRITATING”* *“home care clients are becoming more and more complex, and everything should be documented”* *“wintertime when the roads are icy, poorly plowed, and early spring with poor road conditions”*
	Job discontentment	*“would like to continue to educate myself at regular intervals but it’s difficult to manage with full-time job”* *“little possibility to influence decisions, even though I, for example, know something better than those who decide”* *“It is not always so fun”*
Work community	Negative atmosphere	*“bad atmosphere among the personnel”* *“gossip, mean talk behind your back”*
	Poor engagement	*“poor work ethic among colleagues”* *“if you can't trust your workmates (that the work has been done)”*
***Categories positively affecting occupational self-efficacy belief to continue working until expected retirement age***		
Own health	A resource and a challenge	*“if [my] health stays the same as now, it will be ok”* *“you don’t know whether you are capable until retirement because of the workload”*
	Health supporting activities	*“I must take care of myself so that I am capable”* *“hope to be able to improve [my] work ability”*
Workplace resources	Satisfactory organizational resources	*“support and encouragement from my supervisor”* *“that the employer recognizes age in a positive way”*
	Strong workplace community	*“the support from work colleagues”* *“our fine sense of community in the work team”*
Meaning of the work	Intrinsic motivation	*“[I] like to help people”* *“the feeling of making a difference”* *“love for my own work”*
	Satisfaction from work	*“successful client meetings”* “*[I] enjoy my work tasks”*
Profession in demand	Continuous need of home care	*“there are always sick people”* *“home care is also needed in the future”*
	Skilled in demanding work	*“I don't feel old, I still want to learn new things”* *“master different situations”* *“that the work pace shouldn’t be pushed to the breaking point”*
***Categories negatively affecting occupational self-efficacy belief to continue working until expected retirement age***		
Health-related decline	Current life and health	*“there is no such thing, unless [my] health fails”* *“the pain that is getting worse”*
	Future health challenges	*“that [my] body is not capable of the work”* *“I don't know if I am capable of ‘running’ to the end, probably not”*
Multifaceted work	Demanding work	*“shift work, heavy shifts on the weekends”* *“the importance of anything else but patient contact”* *“ever-increasing demands on knowledge”*
	Imbalance between individual resources and demands	*“elderly people should live at home longer... But less personnel who should manage to get more done”* *“if the workload increases more”* *“too unvaried tasks without challenges”*
Organizational resources	Consequences of lack of resources	*“too few personnel, not competent personnel”* *“permanent fixed-term employment contracts”* *“that we don’t get more personnel when the number of clients increases*
	Unsatisfying leadership	*“constant changes, which have often not been considered to the very end either, you can’t trust things”* *“the worker is of less and less importance”* *“[they] don't listen to the workers”* *“unfair distribution of work, does not apply to everyone”*
Work-related strain	Constantly present rush	*“stress/always a rush during work”* *“too much of a rush with the clients”*
	Imminent threat of stress	*“sometimes sleep poorly; the job is in [my] thoughts...”* *“if… the stress increases more…”*

### Categories positively affecting work motivation

The category “Work environment” included the sub-categories “Satisfaction with colleagues” and “Satisfaction with leadership and organizational resources”. HCNs were motivated by satisfaction with colleagues including good relationships, a sense of belonging in the work community. Well-functioning teamwork was emphasized. It was considered a product of reliable and supportive colleagues with good collaboration skills. HCNs’ motivation was increased by satisfaction with leadership, which included that the labour management trusted the HCNs, and allowed them to take responsibility and influence their work content, routines, work schedules, work quantity, and vacations. Opportunities to perform other tasks for a change or supervise new colleagues were also expressed. Furthermore, home care leader, applying a supportive leadership style, including having a good attitude with workers and good dialogue skills seemed to improve work motivation. High quality work was possible if the following necessary organizational resources were available: appropriate work equipment, enough staff, and good work planning, for example, functioning work schedules and sufficient time to perform client work.

The category “Significance of the work” included the sub-categories “Meaningful work” and “Enriching client meetings”. Home care work itself was a source of work motivation, and was considered important, meaningful, and necessary. An intrinsic motivation to help other people was emphasized; having the willingness, ability, and permission to help clients and make a difference in their everyday life, as well as to bring them happiness. HCNs enjoyed working with the clients and cared for their well-being. Furthermore, home care work entailed recurrent social contacts; HCNs got to know their clients well and felt close to them.

The category “Stimulating challenges” included the sub-categories “Multifaceted work” and “Competence and desire to develop”. Another motivating factor was that home care work comprised both responsibility and freedom. The opportunity to do shift work and the work content were brought up. The feature of home care work; unpredictable, including varying and challenging tasks and client situations, mostly in the clients’ homes, was emphasized. The demanding work, requiring both physical and mental power as well as social skills, seem to increase work motivation, providing it is manageable. According to HCNs, they had the work they desired and were educated for. Additional motivating factors were work experience and professional skills. HCNs trusted themselves and had belief in their skills and competence. They felt motivated by results of work done and success in problem solving. Moreover, they wanted to continue developing their professional skills.

The category “External response” yielded the sub-categories “Recognition from clients and relatives” and “Recognition from colleagues and management”. Positive feedback received externally from others for work done seemed to increase work motivation. Feedback received from the clients appeared as appreciation, praise, and encouragement. A hearty welcome from the clients, who were grateful for getting help and a good care improved motivation. Likewise, recognition from relatives showing their thankfulness for good care. Positive feedback from colleagues as encouragement and displayed satisfaction were found to be important for motivation, as well as home care managers and employers expressing their appreciation.

Moreover, less frequently mentioned responses yielded the categories “Income stability”, “Close relationships and leisure”, and “Sufficient health and energy”.

### Categories negatively affecting work motivation

The category “Organizational work environment” included the sub-categories “Lacking resources” and “Unsatisfactory leadership”. Due to a reduction in financial resources and optimization, worsening care conditions, resources not meeting the needs of care, and inequalities between work teams had a negative effect on work motivation. HCNs stressed inadequate, or lack of, necessary work tools and inappropriate workspaces. Frequent changes in existing responsibilities, new tasks, and ways of working, that often occurred at short notice were perceived as both difficult and burdensome, and caused concern. Additionally, incompetence among the permanent workers and difficulties in finding skilled staff were negatively affecting motivation. Nevertheless, accessible skills among HCNs remained sometimes unused. Moreover, a negative effect on work motivation was a weak leadership. Significantly leaders’ negative attitude towards workers, and their lack of response, support, and appreciation. Insufficient dialogue between workers and leaders was emphasized, including poor information, work instructions and guidance at work. Leaders being inaccessible, escaping their responsibilities or making too high demands, sometimes without adequate follow-ups. Injustice in everyday work, unclear job descriptions, as well as poor work planning and scheduling were accentuated, in addition to HCNs not being involved in the work planning. Further hindrances stressed were unclear criteria and purposes of the home care work, and an overall unsystematic work organization.

The category “Time constraints” generated the sub-categories “Significant rush and stress” and “Negative consequences of time pressure”. According to HCNs, a constant time stress causing a negative strain decreased their work motivation. The work pace was perceived as too high due to time pressure, leading to a considerable and sometimes unreasonable rush. Physical and mental stress were present in everyday work. Among some HCNs, the stress arose periodically, while others considered it a constant and extreme stress. Too many client visits a day and time pressure when driving from one client to another triggered the stress. HCNs were often behind the scheduled time. They experienced less time for completing tasks, and for listening to the clients. Consequently, tasks were left unfinished or undone, with the quality of work suffering and the workload perceived as too hard.

The category “Job characteristics” yielded the sub-categories “A challenging work” and “Job discontentment”. The solitary and highly demanding home care work requiring great responsibility felt burdensome and decreased work motivation. HCNs expressed feelings of uncertainty and being unsatisfied. There were many client visits per day, with often new clients and new tasks. The shift work was perceived as hard, and additionally home care nurses worked a lot of overtime. Little opportunity to influence working hours and schedule, work routines and job tasks, as well as not being involved in decision making were negatively affecting work motivation. An increased amount of administrative work during the last years was perceived as taking too much time from the client work, and some assignments were considered unnecessary. Additionally, the work environment in clients’ homes was often warm and non-ergonomic, and many clients were physically heavy to assist. The weather conditions of the seasons were at times challenging. Many kilometres were driven by car, sometimes in demanding weather and bad road conditions. Alarming was that the already high workload was perceived as increasing, because of a higher amount of work, increasing requirements, and more complex clients with more challenging care needs. HCNs wished to improve their skills, but at the same time they experienced declining levels of energy and willingness. The work became an old habit, and the attitude and the feeling towards work became increasingly negative negatively affecting work motivation.

The category “Work community” yielded the sub-categories “Negative atmosphere” and “Poor engagement”. A poor work community, including a bad atmosphere and negative and tired colleagues, was negatively affecting work motivation. Poor cooperation and conflicts in the working group along with the complaining of others, arrogance, gossip, slander, and jealousy were brought up. In addition, a language barrier between Swedish and Finnish speakers was mentioned, although only by a few HCNs. Lack of commitment and motivation were expressed as poor work ethic and poor activity among the staff; colleagues being lazy, high usage of private mobile phones, and not participating in tasks other than client work. HCNs emphasized that poor responsibility among colleagues when tasks were left undone, and colleagues not helping each other even though needed decreased work motivation. As a result, a loss of confidence in colleagues was seen.

Additional categories decreasing work motivation, although mentioned less, were “Weakened health and energy”, “Feedback from clients and relatives”, “Salary level”, “Staff shortage”, “National controlled decrees” and “Age”.

### Categories positively affecting occupational self-efficacy belief to continue working

The category “Own health” generated the sub-categories “A resource and a challenge” and “Health supporting activities”. Overall, HCNs conveyed their health as important on their belief to continue working, worth investing effort in. The health was both a resource and a challenge. Good health and wellbeing, or manageable health challenges, provided confidence. In contrast, some HCNs felt anxious about their deteriorating health; they perceived worries about their future work ability and voiced the need to prioritize their health before continue working. However, HCNs declared an intention to and an interest in promoting and maintaining their health, by participation in rehabilitation programs, and with appropriate physical exercise, rest and eating habits.

The category “Workplace resources” yielded the sub-categories “Satisfactory organizational resources” and “Strong workplace community”. Well-organized work highly influenced HCNs’ belief to continue working, and included both material resources, good leadership, and good relationships with colleagues. HCNs emphasized having and retaining a sufficient number of both temporary and skilled workers by using continuing employment agreements. The supply of necessary ergonomic tools and possibilities to adapt the clients’ homes for their care needs, as well as job training to cope with demanding clients, provided belief. Furthermore, the availability of modified tasks in line with current work ability, as well as supply of adequate occupational health care service were stressed. A good leader that is supportive, responsive, understanding and encouraging, showing appreciation for efforts made and treating workers equally seem to improve belief to continue working. Moreover, HCNs emphasized the importance of having influence over working hours, workload, work content and opportunities for job rotation. Although age itself was rarely mentioned, HCNs wanted the employer to notice age in a positive way. A strong work community was essential and produced job satisfaction and belief. The HCNs mentioned likable colleagues, with whom they got on well and sometimes developed deeper friendships. In well-functioning work teams, colleagues supported and helped each other. However, sometimes work in another work team in the case of a poor work community could enhance belief to continue working.

The category “Meaning of the work” generated the sub-categories “Intrinsic motivation” and “Satisfaction from work”. A great intrinsic motivation and a strong desire to help and take care of people were positively affecting HCNs’ belief to continue working. HCNs experienced the work as meaningful and highlighted the large amount of people in need of assistance. They stressed feelings of love for their work and stated home care nursing as their dream profession. HCNs liked their clients and the client relations provided social interaction and rewarding meetings. Furthermore, satisfaction with the work tasks and the workplace seem to improve the belief to continue working. However, own work morale was considered essential to continue working.

The category, “Profession in demand” yielded the sub-categories “Continuous need of home care” and “Skilled in demanding work”. Employment stability in the home care sector increased HCNs’ belief to continue working. HCNs emphasized a continuous need of staff, and future demand due to changes in society regarding more people living at home longer. To continue working within the same field and work team also gave a sense of security. Feeling skilled in the multifaceted home care work, with confidence in managing the varying work tasks improved HCNs’ belief to continue working. HCNs saw manageable continued changes and work-related challenges as inspiration, and they wanted to further develop their own professional skills. However, a reasonable mental and physical workload, as well as for a better status for the home care work would further increase HCNs’ belief to continue working.

Furthermore, but in less degree, the categories “Family life and leisure”, “Joy of living and wisdom”, “Income needed” and “Appreciation and feedback” emerged.

### Categories negatively affecting occupational self-efficacy belief to continue working

The category “Health-related decline” yielded the sub-categories “Current life and health” and “Future health challenges”. Simply if the overall health remains; the HCNs did not see any hindrances to continue working until the expected retirement age. However, the current health and balance in life revealed challenges in belief to continue working. HCNs brought up a long list of diagnosed diseases, such as mental health disorders, musculoskeletal diseases, and hypertension. Many reported aches and pains in the back, shoulders, knees, or fingers. Due to the current health limitations, HCNs expressed concerns and uncertainties of whether the current diseases will deteriorate, and about future illnesses. Along with physical decline, high mental strain, insomnia, reduced energy and weakened recovery caused uncertainty about the future. Moreover, the concern of close relatives’ health was also stressed. However, only a few mentioned their ageing as a concern.

The category “Multifaceted work” included the sub-categories “Demanding work” and “Imbalance between individual resources and demands”. HCNs perceived home care work as burdensome, which negatively affected their self-efficacy belief. The shift work was intense and hard, with irregular and inconvenient working hours, and composed of too many shifts without days off. Extended working days, frequent overtime and being asked to work at short notice caused even more aggravation. HCNs stressed an increase in the number of clients, often with more demanding care needs, and yet often less time for caring and assistance. An additional source of discontent lowering self-efficacy belief was the increased number of administrative tasks, sometimes at the expense of client work. Increased work areas also entailed longer work trips and more travel time in the car. Furthermore, an increased requirement on knowledge was experienced, and some HCNs stressed an inadequate knowledge level in relation to the requirements. Moreover, concerns about an imbalance between individual resources and an already heavy, and still increasing, physical and mental workload negatively affected belief to continue working. Care giving in clients’ homes, often with insufficient ergonomic tools, entailed physical heavy workload. The already high and increasing work demands, as well as the uncertainty about future demands and an even greater workload, were mentally challenging. To be constantly flexible at work was also stated to be mentally challenging. Likewise, was dealing with clients and relatives. HCNs sometimes perceived too much responsibility and were concerned about the poor quality of the final home care being provided. In contrast, lack of challenges, personal development, and promotional opportunities also negatively affected belief in being able to continue working in the home care sector.

The category “Organizational resources” yielded the sub-categories “Consequences of lack of resources” and “Unsatisfying leadership”. Lacking resources and economic savings negatively affected HCNs’ belief to continue working and were conveyed as staff reductions, understaffing, hiring freezes, incompetent workers, and the continuous employment of temporary workers. Involuntary team and workplace changes were forced. Additionally, home care work was exposed to frequent and continuous changes due to the organizational changes and fusions of organizations. Many changes were considered irrational, causing either deterioration, or not achieving the expected results, and were mentally stressful and energy draining. Other changes were considered as uncontrollable, and the uncertainty and fear of losing one’s job was revealed. Dissatisfaction with leadership and poor leadership skills were other sources negatively affecting HCNs’ belief to continue working. Lack of support, appreciation, respect, justice, and equality were disclosed, as well as unclear or missing information. HCNs experienced that they sometimes were not listened to, and that their home care managers had inadequate understanding of home care work. Moreover, excessive demands with no resources for realization, as well as poor and unfair work distribution had a negative influence on HCNs’ belief to continue working until retirement age.

The category “Work related strain” generated the sub-categories “Constantly present rush” and “Imminent threat of stress”. A constant rush causing stress reactions was negatively affecting HCNs’ belief to continue working until the expected retirement age. The hurry was an existing companion in the everyday work, perceived to be further increasing, and too hard. Some HCNs wanted to change work, because of the rush. Additionally, concerns about continuous stress decreased HCNs’ self-efficacy belief. Home care nurses expressed reduced stress tolerance, including thoughts about work interrupting their sleep, and that they were worried about future stress aggravated reactions.

In addition, categories with less responses were “Political and national decisions”, “Workplace community”, “Financial reasons”, “Retirement and family life”, “Lack of motivation”, and “Unemployment”.

## Discussion

The aim of this mixed methods study was to explore the ageing HCNs’ work motivation and occupational self-efficacy to continue working until expected retirement age. The findings showed that several categories concurrently affected both work motivation and self-efficacy belief. When the categories were well-functioning, they positively affected both motivation and self-efficacy belief, and when they were insufficient, they negatively affected either or both motivation and/or belief. Our study included participants of 45 years and older and, thus, conveys important knowledge about ageing HCNs. Addressing ageing workers’ needs may help to deal with the lack of skilled health care personnel and to promote their work careers, receiving workers with better well-being and work ability [36].

According to the JD-R model, unmanageable job demands predict health problems [10]. This assumption corresponds with the findings in our study, since the HCNs emphasized the negative impact of high and increasing demands unfavourably affecting both motivation and self-efficacy. Health issues emerged as the most prominent regarding HCNs’ self-efficacy belief. Current health declines, and concerns about future deteriorations, caused uncertainty. Furthermore, the high physical and mental workload experienced, raised concerns regarding health and thoughts to change work field. Despite individual differences, the occurrence of chronic health problems generally increases with age [37]. Decreased work ability in ageing workers can be explained by chronic health problems [38], and an imbalance between individual resources and job demands [39]. Considerations about individual health [40], chronic health problems [38], and, poor health affecting work ability tend to increase an early exit from work [41, 42]. Feelings of being worn-out influences older workers’ decisions on whether to continue working [43, 44]. Self-rated health is however considered a better predictor of extended working life among older health care personnel, compared to diagnosed diseases [44], and is therefore an important predictor of work ability perceptions [45].

An unsatisfying balance between job demands and job resources were revealed in our study in terms of lack of staff and ergonomic tools, unsatisfying leadership, non-supporting work community, increasingly challenging clients, time pressure and stress, lowered quality and tasks left unfinished. Other studies have also revealed the negative effects of high work demands, time pressure, lack of staff and lack of recovery of older assistant nurses’ motivation for full working life [43], the impact of stress, dissatisfaction, managerial style, and support for nurses’ turnover [46]. High mental and physical demands are also mirrored in people’s unwillingness to work in home care [8]. This is highly worrying in a sector with already high and increasing need for staff. There is, therefore, an urgent need to handle unmanageable work demands in a nursing sector that is already stressed [19, 43]. Work organizations are responsible to arrange work in a way that supports workers. Organizations must respect and listen to the workers and address their needs [47]. To retain HCNs in working life, reasonable workload, flexible work scheduling, and autonomy and participation in decision making work have been suggested [19]. Increasing the number of staff and improving recovery opportunities during work shifts and work-life balance have also been stressed to improve working conditions [43]. However, home care managers’ lack of understanding both of HCNs’ chronic health issues and the implementation of home care have also recently been emphasized [8, 43, 46]. HCNs wish for more knowledge about practical home care work from home care managers and decision makers [8].

To influence the high demands addressed and take actions to make a sustainable working life for the ageing HCNs, the leaders and the work organizations must be involved to succeed. They have the authority to make changes that on a long term influence the workers’ ability to remain in working life [40, 48]. Interventions enhancing working life must pay attention to the complexity of work life [40], and be performed in parallel on the different levels [14, 40, 49]. The swAge model [40] is a new concrete and multi-level tool for managers and organizations, as well as for workers, to both identify risks and develop work issues that contribute to a sustainable working life, throughout the whole working career. Areas important to the HCNs in our study fits into the model and need to be considered by the leaders and the organization; the health, and its relation to physical and mental work demands, leadership, development, meaningful work, workplace community, and social support [40]. However, societal demands on increased workplace measures might be needed to further press organizations to implement strategies to retain older workers in work life [40, 50, 51].

The JD-R model proposes that persons having high job resources have higher motivation and can better cope with high job demands [10]. For example, HCNs’ job resources, such as autonomy, social support, performance feedback, and opportunities for professional development, have been found to buffer the relationship between job demands and burnout [52]. The importance of meaningfulness and job satisfaction for ageing workers [21, 46, 53] and for HCNs to remain employed [19] has been highlighted in several studies. Previous research has found that perceived job satisfaction [44] and meaningful tasks [54] affect self-rated health. Moreover, perceived meaning of work might also moderated the negative effect of age on work ability [11]. Similar to these findings, HCNs in our study emphasized the meaningfulness and satisfaction, both increasing work motivation and self-efficacy belief to continue working. The HCNs enjoyed their work. They cared for the clients, desired to help and make a difference in people’s lives, which also contributed to rewarding client meetings. Helping others and making a difference have both been found meaningful [8, 43] and important for HCNs to continue working [19]. The relationship between staff and clients has also been shown to be reciprocal; providing for someone gave satisfaction [43] and a sense of fulfilment and provided personal development [55]. In a recent meta-analysis, job satisfaction was revealed to be critical in relieving the shortage of nursing staff, and both job satisfaction and intrinsic motivation could be improved through building professional and workplace meaning by value-driven strategies [21].

Another job resource revealed in our study was the high value of healthy and supporting relations in the workplace. Home care managers engaging in dialogues with HCNs actively expressing feedback and support, appreciating their great knowledge and allowing them to influence their work, facilitated HCNs’ self-efficacy belief to continue working. Likewise, HCNs emphasized that support and feedback from colleagues, and a wealthy work community is important. These findings correspond with previous research in which support from colleagues and leaders were found to be important for workers’ motivation [43] and well-being [8, 40, 53], and for HCNs to remain employed [19]. Despite the travelling character of home care work, it is important that employers make opportunities for team and informal meetings among HCNs to strengthen the social relationships [19]. Working in smaller teams has been suggested to support both workplace well-being and the quality of home care, since it can decrease the number of frequently new clients [8], as well as provide well-functioning teamwork and collaboration, as highlighted in our study.

According to the JD-R model, positively valued demands might have a motivational potential since enough job resources are available [10, 56, 57]. In our study, autonomy, flexibility, and the multifaceted character of work were positively perceived in conditions of balanced resources and negatively in conditions of unmanageable demands. These findings correspond with the results in previous research, where autonomy, flexibility and decision authority contributed to HCNs’ intention to continue working [19]. The HCNs in our study also felt skilful in their work and were strengthened using their various skills and succeeding in challenges. Although a reduction in energy was perceived as a hindrance for one’s own development, HCNs still wanted to face new challenges and develop in their work. Previous research has shown that the use of knowledge and opportunities to learn positively affect workers’ self-efficacy [58, 59], motivation and intentions to stay in working life [19, 43, 53]. Thus, HCNs should be offered opportunities for continuous education and development [13, 19, 40, 45, 59, 60], as well as to influence the improvement of home care work.

The JD-R model suggests that personal resources such as self-efficacy can play similar roles as job resources [10]. Thus, self-efficacy belief is effective in promoting health behaviour change [49, 61]; crucial for both motivation [49] and work ability among HCNs [14]. People with high self-efficacy believe that good things will happen and have control over their situation [10]. They have the power to produce desired changes by their actions and will translate health knowledge into health enabling practices with minimal support [49]. They have the incentive to act and persevere in the face of difficulties. Contrary, people with self-doubts about their efficacy, or belief that health habits are beyond their control, need considerable support and guidance to strengthen their perseverance, and to build progressive success through mastery experiences by overcoming challenging obstacles [49]. Thus, the level of individual support needed depends on the level of the workers’ self-efficacy belief. Self-efficacy can be developed by mastery experiences by overcoming challenging obstacles, modelling by seeing peers succeed, positive feedback, and group support and encouragement, until one starts believing in one’s capabilities [49, 62, 63]. However, even when workers have succeeded in performing challenging tasks, a required effort to get work tasks done might decrease self-efficacy beliefs if the workers feel unsure about whether they would be able to produce the same effort again [63]. Every task left unfinished or done with poor quality, or every time the HCNs struggle to cope with lifting or meeting care needs, will affect self-efficacy belief. It is therefore crucial to support every step that may improve a feeling of success.

Both health [64], and work ability can be viewed as movements on a continuum, from perfect health or work ability to complete disability, and where different degrees of disability is a normal part of working life [65] Accordingly, to some degree and on some level it is possible to increase health during the lifetime, through a human being’s potential to continuously learn, develop and renew self [66]. Meaning and intrinsic needs reflect the desire, belief in one’s capabilities, and efforts taken, to maintain work health. Healthy habits and behaviour facilitate health; meaningful and sustainable health can be obtained only when a person becomes aware of and actively follows the inner values leading to health [66]. Furthermore, a sustainable working life presupposes that the worker is treated with dignity, respect, and trust in a supporting work community [66, 67].

### Methodological considerations

A strength in our study was the use of a cross-sectional survey, since the prevalence of multiple outcomes could be measured, and many participants could be reached at the same time [68]. All HCNs in the chosen region, aged 45 years and older, were invited, and were considered as representatives of ageing workers in the home care sector. However, their experience of home care work differed, and this may have affected the answers regarding perceived challenges and resources. Another strength of this study was the use of mixed methods, including both qualitative and quantitative analysis of the research data. The manifest qualitative analysis provided a deeper knowledge and understanding of the issues [30, 35]. Moreover, the quantitatively measured frequencies of utterances gained reliability since the focus of HCNs’ experiences was acknowledged [30]. Nevertheless, the cross-sectional design, describing the phenomena at one specific time, limited the ability to derive causal relationships [68].

A limitation when using a questionnaire is a risk of high numbers of dropouts. The response rate was 51%, which could imply a possible outcome bias. The close managers who administered the survey decided whether a paper-based and/or web-based questionnaire was most suitable in their team. The response rate was slightly lower among the web-based respondents. Hence, some none-responding HCNs might have answered a paper-based survey. Moreover, the research data consisted of open-ended questions. The answers were often short, which despite manifest analysis, might have caused some interpretation bias [35]. Therefore, we returned repeatedly to the meaning units as well as all the respondent’s answers to check the entirety and the meaning [30, 35]. The response rate of the open-ended questions among the answering respondents was high (86–97%, *n* = 202–227 for each question), although the open-ended questions were placed last in quite a large-scaled survey. This indicated that HCNs had an interest to articulate their work motivation and belief in their capabilities to continue working. Another strength was that the findings not only highlighted what motivates HCNs to perform work tasks, but also an intrinsic motivation to care, reflected by internal feelings of care for clients and a deeper meaning of the work.

The HCNs’ responses were either in Swedish or Finnish language. None of the researchers had Finnish as their mother tongue, although two of them mastered Finnish well. In the case of linguistic doubts, a person with Finnish as their mother tongue, also skilled in health care, was consulted. A strength was that no linguistic misunderstandings appeared in the pilot testing of the questionnaire. The researchers’ pre-understanding consisted of long experience in health care, occupational health service and rehabilitation as well as experience in qualitative and quantitative research. To minimize the pre-understanding, the qualitative data analysis followed a structured procedure in several steps [30, 35]. Dependability was established by repeatedly going back to the encoding, verifying the encoding to the meaning units and the open-ended answers, and checking the reliability of the categories [30, 35]. Notes about the encoding were made throughout the analysis process. To increase conformability, triangulation between researchers was used. Quotations were used to support authenticity in presented categories (Table 2) [30]. Moreover, our study addressed the explicit views of the ageing HCNs, in the context of both rural and urban areas, including home care services conducted by municipalities, cities, and social- and health services consortiums. The categories that emerged through manifest content analysis were considered logical and answering the purpose of the study. The findings from our study can likely be transferred to other Nordic countries, due to their similarities in home care service [6]. However, educational requirements for staff may differ in different countries. Despite home care including distinct elements concerning care in clients’ homes; the results might also be transferable to ageing workers in other nursing contexts [30, 35].

### Practical implications

The results of this study convey important understandings of the ageing HCNs’ views of work motivation and occupational self-efficacy belief to continue working until expected retirement age. By supporting HCNs’ health and emphasizing the meaningfulness and positive aspects of the work, offering overcoming challenges and learning opportunities, the HCNs’ work motivation and self-efficacy belief to continue working can be strengthened. This information could be used by home care managers, organizational management, occupational health personnel, safety personnel, and by HCNs themselves, to consciously reflect on and develop a sustainable work life for ageing HCNs. Addressing the needs of ageing HCNs could also help employers deal with the lack of workers in the home care sector. In turn, employers could receive more workers with better well-being and with possibly longer work careers.

## Conclusions

Home care work was perceived as meaningful and stimulating, and through highlighting these positive aspects together with strengthening the work community, both work motivation and occupational self-efficacy belief to continue working might be facilitated. The perceived health highly affected the self-efficacy belief to continue working, emphasizing that work related physical and mental demands must be balanced against the workers’ resources, in combination with a healthy leadership. The results convey information to home care managers, organizational management, safety staff, labour unions and the ageing workers about important issues where intervention is needed, as well as that create good conditions, to make a sustainable working life for ageing HCNs, and thereby improve the ability to retain HCNs, as well as attract new staff to home care.

## Supplementary Information

**Additional file 1.**

## Data Availability

The data is not publicly available but could be requested from corresponding author after ethical approval to take part of the dataset.

## Abbreviation

**HCNs**: Home care nurses
