# Supplementary material for: Work motivation and occupational self-efficacy belief to continue working among ageing home care nurses: a mixed methods study
Source: BMC Nurs. 2022 Jan 27;21:31. doi: 10.1186/s12912-021-00780-3 (PMC8793160; doi:10.1186/s12912-021-00780-3)
Supplement: Supplementary file 1 — Additional file 1. [file 12912_2021_780_MOESM1_ESM.docx]

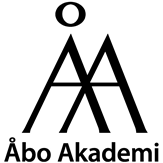
Additional file 1. English translation of the open-ended questions

Work motivation

**Name three things that give you work motivation**

1. ­­­­­­­­­­­­­­­­­­­­­­­­­­_____________________________________________________________________________
2. _____________________________________________________________________________
3. _____________________________________________________________________________

**Name three things that impact negatively on your work motivation**

1. ­­­­­­­­­­­­­­­_____________________________________________________________________________
2. _____________________________________________________________________________
3. _____________________________________________________________________________

Belief in own capabilities to continue working

**Name three things that make you believe that you can work until expected retirement age**

1. _____________________________________________________________________________
2. ___________________________________________________________________________­­__
3. _____________________________________________________________________________

**Name three things that impact negatively on your belief that you can work until expected retirement age**

1. _____________________________________________________________________________
2. _____________________________________________________________________________
3. _____________________________________________________________________________
